# A predictive model and nomogram for coronary artery injury in Kawasaki disease based on laboratory indicators: a retrospective study

**DOI:** 10.3389/fped.2026.1777432

**Published:** 2026-04-30

**Authors:** Yanyan Li, Zhiqing Chen, Xiaoyan Wang, Chaolong Zheng, Ziyang Cui, Sisi Cheng, Limin Chu, Changjun Ren, Guiling Liu

**Affiliations:** Department of Pediatrics, The First Hospital of Hebei Medical University, Shijiazhuang, China

**Keywords:** coronary artery lesion, Kawasaki disease, laboratory parameters, nomogram, predictive model

## Abstract

**Background:**

To explore the changes in various laboratory parameters in children with Kawasaki disease, analyze their correlations with complete and incomplete Kawasaki disease as well as coronary artery lesions and non-coronary artery lesions, and establish prediction models and nomograms.

**Objective:**

Incomplete Kawasaki disease (IKD) is prone to misdiagnosis, and coronary artery lesion (CAL) represents its severe complication. This study aimed to explore the predictive value of routine laboratory indicators for typical/incomplete KD and CAL, and to construct reliable predictive models and nomograms, thereby providing quantitative tools for early screening and risk stratification in primary clinical practice

**Methods:**

A total of 131 children with confirmed Kawasaki disease (KD) from January 2023 to June 2025 were retrospectively enrolled, who were assigned to the TKD group (*n* = 95) and IKD group (*n* = 36), as well as the coronary artery lesion (CAL) group (*n* = 39) and non-CAL (NCAL) group (*n* = 92). Univariate and multivariate logistic regression analyses were applied to screen out independent influencing factors. The performance of the predictive models was evaluated using receiver operating characteristic (ROC) curve, calibration curve and decision curve analysis (DCA). Bootstrap resampling was adopted for internal validation of the models, and visual nomograms were constructed accordingly.

**Result:**

Total protein (TP) was the only independent factor for differentiating typical from IKD; hypoalbuminemia, hyponatremia, and elevated lactate dehydrogenase (LDH) were identified as independent risk factors for KD complicated with CAL, with hypoalbuminemia being the strongest predictor (OR = 0.783, *P* = 0.001). The area under the curve (AUC) of the predictive model for TKD was 0.762, and that of the CAL predictive model was 0.790. Both models showed good calibration and positive clinical net benefit, and the corresponding nomograms enabled rapid individualized quantitative risk prediction.

**Conclusion:**

This study confirmed the clinical value of routine laboratory indicators in phenotypic differentiation of KD and risk prediction of CAL. The constructed predictive models and visual nomograms feature convenient detection and simple operation, which can provide practical references for the early precise diagnosis and treatment of KD and the prevention and control of CAL risk, and are particularly suitable for primary medical institutions with limited diagnostic resources.

## 1 Introduction

Kawasaki disease (KD) is an acute systemic vasculitis syndrome predominantly affecting children under 5 years of age, with an elusive etiology and pathogenesis to date (1, 2). As the leading cause of acquired heart disease in pediatric populations globally, its incidence has risen gradually in recent decades (1, 3), and its most severe complication is coronary artery injury (CAL), including coronary artery dilation and aneurysm formation. CAL not only impairs the short-term prognosis of KD patients but also markedly increases the risk of ischemic heart disease in adulthood (1, 2), posing a persistent threat to children's long-term cardiovascular health.

Classic (complete) KD is diagnosed based on persistent fever ≥5 days plus at least four of five characteristic clinical manifestations: bilateral non-exudative conjunctival congestion, oropharyngeal mucosal congestion with strawberry tongue and lip fissuring, polymorphous rash, peripheral extremity indurative edema and membranous desquamation, and non-suppurative cervical lymphadenopathy (1, 4). However, 10%–20% of KD cases present with an incomplete (atypical) phenotype (3, 5), defined as persistent fever with only 2–3 typical manifestations. The non-specific clinical features of incomplete KD create great challenges for early clinical identification, and delayed diagnosis and treatment of this subtype are associated with a higher risk of CAL (5, 6), making it a critical unmet clinical need in KD management.

Currently, identifying objective, easily detectable laboratory parameters to assist KD diagnosis, assess disease severity, and predict CAL risk is a key focus in pediatric cardiovascular research (7). Previous studies have validated the clinical value of multiple laboratory indicators in KD, such as C-reactive protein (CRP) and erythrocyte sedimentation rate (ESR) for systemic inflammation, and N-terminal pro-brain natriuretic peptide (NT-proBNP) for myocardial involvement and intravenous immunoglobulin (IVIG) resistance (4, 6). However, single indicators are limited by suboptimal sensitivity and specificity, failing to comprehensively evaluate KD phenotypes and CAL risk. Comprehensive analysis of routine laboratory indicators, exploration of their correlations with complete/incomplete KD and CAL, and construction of quantitative predictive models are therefore essential for early identification of incomplete KD and CAL risk stratification (5, 8).

Routine hematological and biochemical indicators are widely available in primary medical institutions due to convenient detection, low cost, and rapid reporting, yet their potential for early KD screening and risk assessment remains underutilized. Here, we retrospectively analyzed changes in multiple routine laboratory indicators in 131 confirmed pediatric KD cases, and systematically investigated their correlations with complete/incomplete KD phenotypes and CAL occurrence. We aimed to establish a laboratory-based evaluation system and construct reliable predictive models with visual nomograms, providing an objective, quantitative, and user-friendly tool for early screening, phenotypic differential diagnosis of KD, and CAL risk prediction in primary clinical settings. This study is expected to improve the early diagnosis rate of incomplete KD, optimize CAL risk stratification and clinical decision-making, reduce missed diagnoses and unnecessary medical interventions, and ultimately enhance the short- and long-term prognosis of children with KD.

## 2 Objects and methods

### 2.1 Research object

A total of 131 children diagnosed with KD at the First Hospital of Hebei Medical University from January 2023 to June 2025 were selected as the study group. Among them, there were 95 cases of typical Kawasaki disease children and 36 cases of incomplete Kawasaki disease children. Among the Kawasaki disease children, 39 cases had coronary artery injury and 92 cases had non-coronary artery injury. This study has been approved by the Ethics Committee of our hospital.

### 2.2 Diagnostic criteria

#### 2.2.1 Typical Kawasaki disease

Fever for ≥5 days (a necessary condition), plus at least 4 of the following 5 main clinical features. Five main clinical features: conjunctival congestion of both eyes: bilateral, non-exudative, painless. Lip and oral changes: Red, chapped and bleeding lips, strawberry tongue, diffuse congestion of the oral and pharyngeal mucosa. Polymorphic rash: Mainly maculopapular rashes on the trunk and limbs, without blisters or crusts. Changes at the extremities: Acute stage: Erythema on the palms and soles, hard edema on the hands and feet. Recovery period: Membranous peeling at the tips of fingers (toes). Non-suppurative cervical lymph node enlargement: often unilateral, with a diameter >1.5 cm (4, 8).

#### 2.2.2 Incomplete Kawasaki disease

Fever for ≥5 days, but only meeting 2 or 3 major clinical features, coronary artery lesions detected by echocardiography (such as Z value ≥2.5) can be diagnosed, or there are ≥3 abnormal auxiliary laboratory indicators: Albumin ≤3.0 g/dL, anemia (age-appropriate), elevated alanine aminotransferase (ALT), platelet count ≥450 × 10⁹ /L on the 7th day of the disease course, white blood cell count ≥15 × 10⁹ /L, urine white blood cell count ≥10/high-power field (excluding urinary tract infection) (4, 8).

#### 2.2.3 Coronary artery injury in Kawasaki disease (9)

Coronary artery injury is defined as a diagnosis where the local inner diameter of the coronary artery is dilated by ≥1.5 times compared to the adjacent area or the Z value of the coronary artery inner diameter is ≥2.0.

### 2.3 Inclusion and exclusion criteria

**Inclusion criteria for Kawasaki disease:** (1) Meeting the diagnostic criteria for typical Kawasaki disease, incomplete Kawasaki disease, and coronary artery injury in Kawasaki disease; (2) First diagnosed as KD; (3) No immune-related drugs or hormone therapy had been received before admission. **Exclusion criteria**: (1) Accompanied by congenital heart disease, congenital malformations, autoimmune diseases, chromosomal abnormalities, heart valve disease and genetic metabolic diseases, etc. (2) Accompanied by other acute or chronic infections; (3) Underdeveloped vital organs (such as the liver and kidneys); (4) Previous history of coronary artery disease. (5) Abnormal coagulation function, etc.

### 2.4 Methods

#### 2.4.1 Software and methods

Statistical analysis of the results was performed using SPSS 27 software. Categorical variables were presented as frequencies (percentages) and analyzed by the chi-square test; continuous variables were expressed as mean ± standard deviation ($\bar{x}\pm s$). After normality test, most samples did not follow a normal distribution, and thus the non-parametric independent samples test was used for comparison. Subsequently, based on clinical laboratory indicators, R software was applied to construct a logistic regression model for the diagnosis of coronary artery lesions in patients with Kawasaki disease. Bootstrap resampling was used for internal validation of the model to systematically evaluate the predictive performance, calibration, and clinical utility of the model. A *P*-value < 0.05 was considered statistically significant. Additionally, nomograms were plotted using R software.

#### 2.4.2 Model construction and evaluation

##### 2.4.2.1 Model construction

Taking the presence of typical symptoms as the dependent variable and the clinically significant laboratory indicators identified in the difference analysis as independent variables, a multivariate logistic regression model was constructed.

##### 2.4.2.2 Model performance evaluation metrics

The performance of the model was evaluated from three core dimensions: discrimination, calibration, and clinical utility. Meanwhile, the Brier score was calculated to quantify the model's prediction error.

Discrimination metrics: Area under the receiver operating characteristic curve (AUC), accuracy, sensitivity (true positive rate), and specificity (true negative rate).

Calibration metrics: Calibration intercept and calibration slope. Under the ideal condition, the intercept is 0 and the slope is 1, which reflects the consistency between the model-predicted probability and the actual occurrence probability. The Hosmer-Lemeshow test was also performed, with a *P*-value > 0.05 indicating good model calibration.

Clinical utility: The net benefit of the model at different risk thresholds was assessed via decision curve analysis, which reflects the application value of the model in clinical practice.

Prediction error: Brier score, which ranges from 0 to 1; a value closer to 0 indicates a smaller prediction error of the model.

##### 2.4.2.3 Internal model validation

Bootstrap resampling was used for internal model validation to correct for overfitting bias, with a total of 1,000 resampling iterations. The specific steps were as follows: Bootstrap samples with the same sample size as the original data were randomly drawn with replacement from the original dataset; a logistic regression model was fitted on each Bootstrap sample; the apparent performance of the model on the Bootstrap samples and the validated performance on the original data were calculated separately, with the difference between the two defined as the optimism bias; the corrected model performance was obtained by subtracting the average optimism bias from the apparent performance of the original model; the 95% confidence interval (95% CI) of each performance metric was calculated using the percentile method to evaluate the statistical stability of the metrics. In the meantime, supplementary validation of the model was conducted to obtain the corrected values of statistical parameters including Dxy and *R*^2^.

##### 2.4.2.4 Model visualization

Four types of visual charts were constructed to intuitively display the model performance: ① ROC curve: comparing the apparent AUC and Bootstrap-corrected AUC to demonstrate the model's discrimination ability; ② Calibration curve: combining the logistic calibration line, smooth curve and grouped scatter points to show the consistency between the predicted probability and the actual disease probability, with the results of the Hosmer-Lemeshow test labeled; ③ Nomogram: converting the model regression coefficients into scores to realize individualized quantitative prediction of the risk of Kawasaki disease; ④ Decision curve: comparing the net benefits of three strategies (model-based intervention, universal intervention, and no intervention) to determine the clinically applicable risk threshold of the model.

## 3 Results

### 3.1 Typical Kawasaki disease group and incomplete Kawasaki disease group

#### 3.1.1 Univariate analysis of typical Kawasaki disease group and incomplete Kawasaki disease group

A total of 131 samples were included, among which 95 samples were from the typical group and 36 samples were from the incomplete group. The general information of the samples and the blood test data are shown in Tables 1, 2 below: Through non-parametric independent sample testing, the results show that there is no difference in age, gender, height and BMI between the typical group and the atypical group in the comparison of general information, but there is a difference in weight. There were significant differences between the two groups in the laboratory indicators of BASO, MONO, Hb, CK, K, Na, Cl, TP and ALB. Compared with the atypical group, the typical group had lower BASO, lower MONO, lower Hb, lower CK, lower K, higher Na, higher Cl, lower TP and lower ALB.

**Table 1:** Univariate analysis results of the typical Kawasaki disease group and the incomplete Kawasaki disease group.

Indicator	Overall	IKD (*n* = 36)	TKD (*n* = 95)	Statistics	*P*
General information					
Age (m)	34.9 ± 29	29.17 ± 26.78	37.08 ± 29.64	−1.643	0.100
Height (cm)	92.23 ± 22.65	87.31 ± 23.65	94.09 ± 22.09	−1.826	0.068
Weight (Kg)	15.42 ± 12.41	13.19 ± 6.8	16.26 ± 13.9	−1.994	0.046
BMI (kg/m^2^)	16.35 ± 2.37	16.61 ± 1.89	16.24 ± 2.54	−0.864	0.388
Blood routine					
WBC (×10^−9^/L)	15.01 ± 5.9	16.43 ± 8.57	14.46 ± 4.4	−0.549	0.583
NEU%	61.38 ± 17.61	58.92 ± 19.71	62.33 ± 16.74	−1.092	0.275
LYM%	27.66 ± 14.65	28.39 ± 16.51	27.38 ± 13.95	−0.339	0.735
EO%	2.45 ± 2.94	2.47 ± 2.96	2.44 ± 2.95	−0.236	0.813
BASO%	0.38 ± 0.47	0.38 ± 0.32	0.38 ± 0.52	−2.605	0.009
MONO%	7.97 ± 5.07	9.55 ± 5.06	7.35 ± 4.97	−3.049	0.002
PLT (×10^-9^/L)	340.33 ± 109.55	348.08 ± 133.8	337.32 ± 99.26	−0.139	0.889
Hb (g/L)	115.56 ± 35.21	121.28 ± 64.68	113.34 ± 10.76	−2.058	0.040
RBC (×10^-12^/L)	4.66 ± 3.81	5.06 ± 5.02	4.51 ± 3.25	−1.208	0.227
Inflammatory indicators					
CRP (mg/L)	72.29 ± 50.56	77.06 ± 54.86	70.48 ± 49.01	−0.443	0.657
PCT (ng/mL)	1.44 ± 2.46	1.86 ± 3.84	1.28 ± 1.68	−0.826	0.409
Biochemical indicators					
ALT (U/L)	45.66 ± 60.98	26.14 ± 18.4	53.65 ± 70	−1.387	0.165
AST (U/L)	48.65 ± 39.18	46.61 ± 29.88	49.48 ± 42.53	−0.501	0.616
CK (U/L)	68.38 ± 49.52	74.94 ± 43.99	65.7 ± 51.61	−2.098	0.036
K (mmol/L)	4.47 ± 0.64	4.66 ± 0.57	4.39 ± 0.66	−2.648	0.008
Na (mmol/L)	134.61 ± 2.96	133.76 ± 3.07	134.95 ± 2.86	−1.966	0.049
Cl (mmol/L)	100.35 ± 3.75	99.34 ± 2.93	100.77 ± 3.97	−2.376	0.018
TP (g/L)	65.19 ± 8.34	68.59 ± 10.81	63.79 ± 6.68	−3.898	<0.001
ALB (g/L)	38.11 ± 5.77	38.74 ± 8.7	37.85 ± 4.04	−2.455	0.014
ALP (U/L)	193.1 ± 92.72	196.61 ± 126.39	191.67 ± 75.63	−0.234	0.815
LDH (U/L)	301.2 ± 76.82	314.08 ± 74.84	295.93 ± 77.41	−1.687	0.092

**Table 2:** Results of gender univariate analysis in the typical Kawasaki disease group and the incomplete Kawasaki disease group.

Gender	Total (%)	IKD (%)	TKD (%)	Statistics	*P*
Male	81 (61.83)	19 (52.78)	62 (65.26)	1.724	0.189
Female	50 (38.17)	17 (47.22)	33 (34.74)		
Total	131	36	95		

#### 3.1.2 Independent influencing factors

The blood test indicators that showed significant differences in the above non-parametric independent sample tests were included in the multivariate logistic regression at one time. The results are shown in Table 3 as follows: The indicator TP was significant in the multivariate logistic regression and was an independent influencing factor of typical Kawasaki disease.

**Table 3:** Multivariate logistic regression analysis of typical Kawasaki disease group and incomplete Kawasaki disease group.

Indicator	B	Std.Error	Wald	Sig.	Exp(B)	95% CI for EXP (B)	
						lower	Upper
BASO	0.221	0.503	0.193	0.660	1.247	0.466	3.340
MONO	−0.044	0.044	1.006	0.316	0.957	0.878	1.043
HB	−0.007	0.007	1.089	0.297	0.993	0.981	1.006
CK	−0.005	0.004	1.340	0.247	0.995	0.987	1.003
K	−0.427	0.359	1.414	0.234	0.652	0.323	1.319
Na	0.045	0.065	0.473	0.491	1.046	0.921	1.188
Cl	0.032	0.078	0.174	0.677	1.033	0.887	1.203
TP	−0.089	0.035	6.491	0.011	0.915	0.854	0.980
ALB	0.023	0.041	0.303	0.582	1.023	0.943	1.109

#### 3.1.3 Model construction formula

Taking the presence of typical symptoms as the dependent variable and the above 9 clinically significant laboratory indicators identified in the difference analysis as independent variables, a multivariate logistic regression model was constructed. The model formula was as follows: Group ∼ BASO + MONO+ HB + CK + K + Na + Cl + TP + ALB.

#### 3.1.4 Apparent predictive performance of the model

The apparent area under the receiver operating characteristic curve (AUC) of the model was 0.762, indicating a moderate discriminatory ability of the model for samples with typical and incomplete symptoms. The overall apparent accuracy of the model was 0.746, with a sensitivity of 0.930 and a specificity of 0.306, which demonstrated that the model had an excellent ability to identify positive samples with typical symptoms and could effectively reduce false-negative misdiagnoses, yet its ability to identify negative samples was relatively weak, with a certain possibility of false-positive diagnoses. The apparent calibration of the model reached an ideal state, with a calibration intercept of 0.000 and a calibration slope of 1.000, suggesting that the model-predicted probability was consistent with the actual occurrence probability of typical symptoms in the original dataset. The *P*-value of the Hosmer-Lemeshow test was >0.05, which further verified the good apparent calibration performance of the model with no significant prediction bias. After calibration with 1,000 Bootstrap resampling iterations, the AUC of the model decreased to 0.679, with an overall accuracy of 0.699, a sensitivity of 0.896 and a specificity of 0.237. All performance metrics showed a slight decrease compared with the apparent results, indicating a mild overfitting of the model, while the model still maintained a moderate discriminatory ability and good internal stability overall. The calibrated calibration intercept of the model was 0.369 and the calibration slope was 0.524; the deviation of the slope from the ideal value of 1 suggested a potential bias in the amplitude of predicted probability of the model in external samples, meaning that the fluctuation range of the predicted probability needs further calibration, yet the model still possessed a certain degree of calibration reliability overall. The detailed values are shown in Table 4.

**Table 4:** Apparent predictive performance of the model in the TKD and IKD groups.

Model performance	AUC	Accuracy	Sensitivity	Specificity	Calibration intercept	Calibration slope
Apparent results	0.762	0.746	0.930	0.306	0.000	1.000
Calibrated results	0.679	0.699	0.896	0.237	0.369	0.524

#### 3.1.5 ROC curve analysis

**Figure 1:**
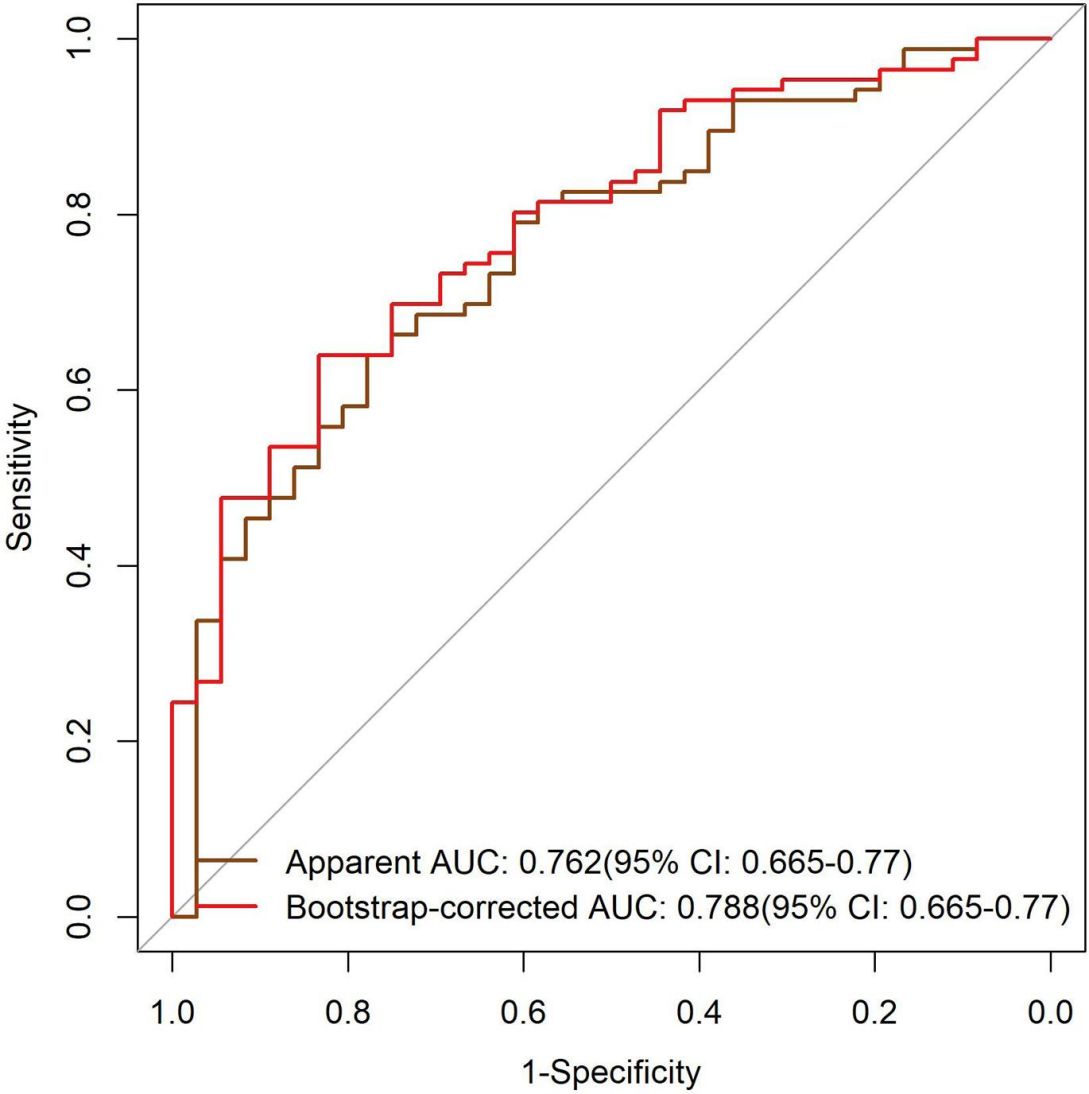
ROC curves of the TKD group and the IKD group.

The results of the ROC curve showed that the apparent AUC of the model was in the range of 0.7–0.8, and the calibrated AUC was in the range of 0.6–0.7. Although the discriminatory ability slightly decreased after calibration, the model still possessed a moderate level of discriminatory ability for samples. The 95% confidence interval of the AUC index was relatively narrow, which confirmed the statistical reliability of the model's discriminatory index and could provide a quantitative reference for the risk screening of typical symptoms (Figure 1).

#### 3.1.6 Calibration curve analysis

**Figure 2:**
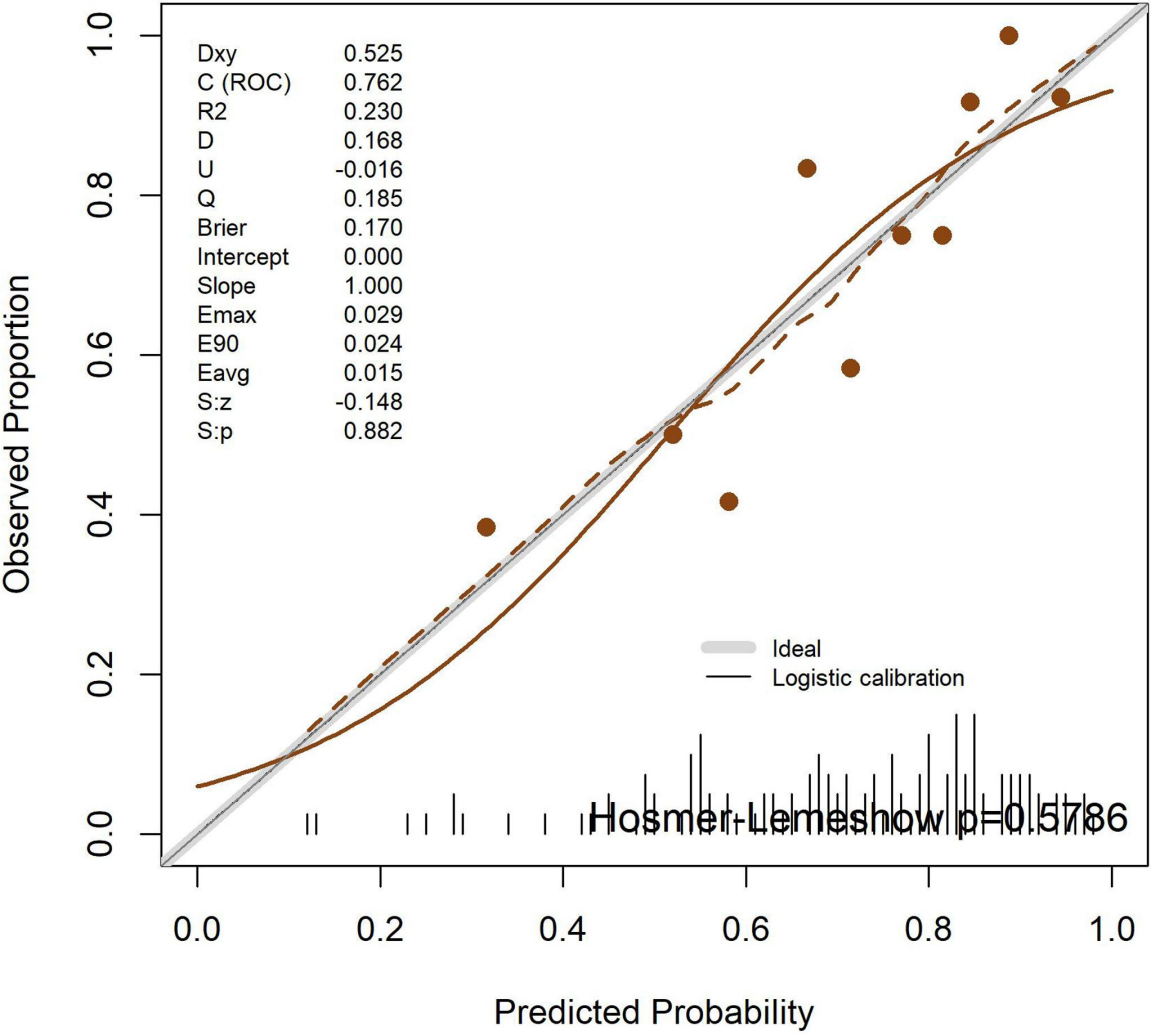
Calibration curves of the TKD group and the IKD group.

The calibration curve results showed that the apparent calibration line of the model coincided with the ideal reference line (intercept = 0, slope = 1), which was consistent with the apparent calibration indicators. After calibration, the calibration line deviated slightly from the ideal line, which was consistent with the calibration parameters after Bootstrap correction. After grouping the predicted probabilities by quantiles, the scatter points of the mean predicted probabilities and the mean actual occurrence probabilities of typical symptoms in each group were generally distributed around the ideal line, suggesting that the overall calibration performance of the model was acceptable and the consistency between the predicted probabilities and the actual occurrence probabilities was favorable (Figure 2).

#### 3.1.7 Decision curve analysis

**Figure 3:**
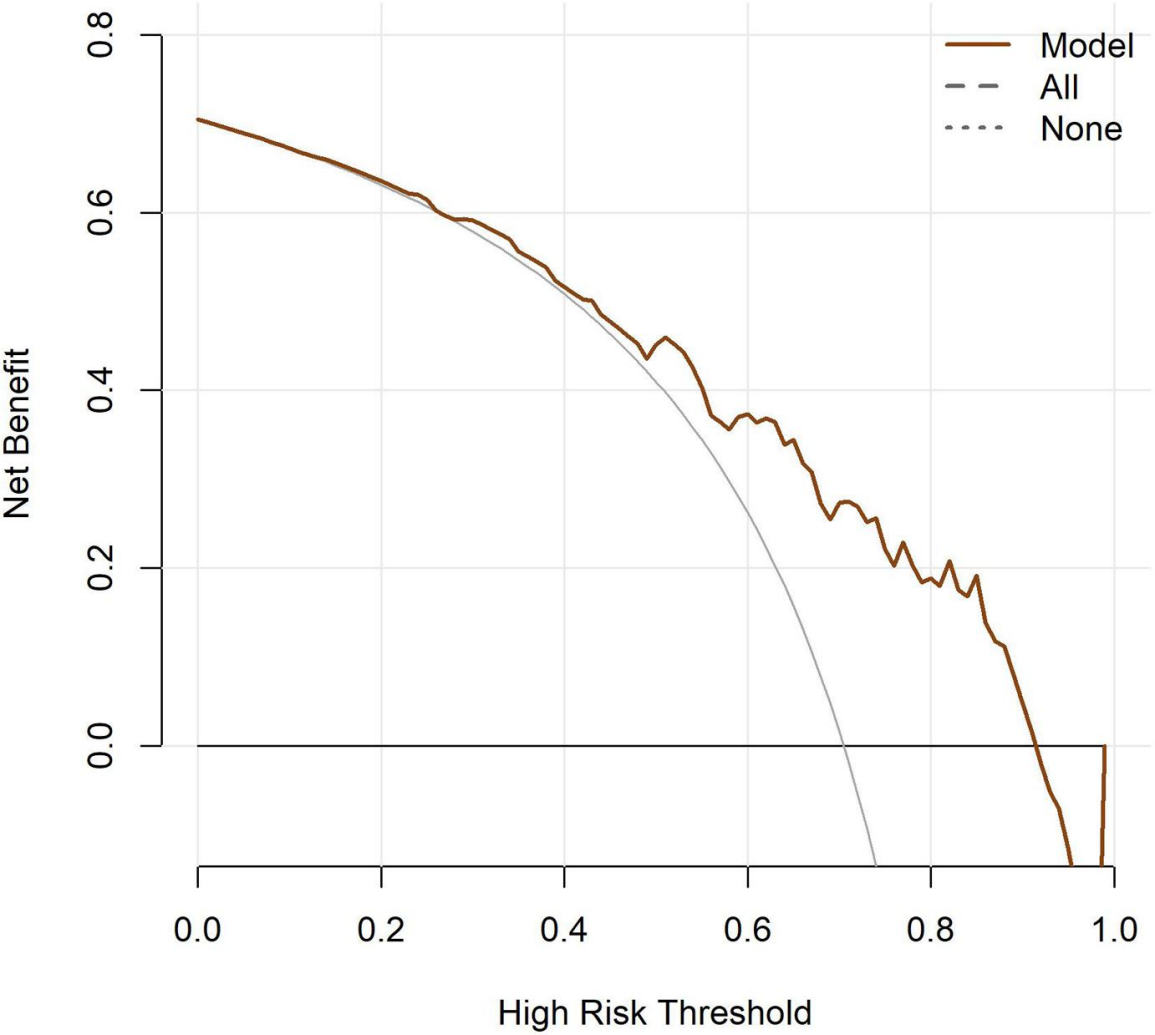
Decision curves of the TKD group and the IKD group.

The decision curve analysis showed that within most risk thresholds for typical symptoms, the net benefit of the model constructed in this study was significantly higher than that of the two extreme strategies: "treat all" and "treat none". This indicated that the clinical application of this model could effectively assist clinicians in identifying high-risk populations with typical symptoms, reduce unnecessary medical interventions, and exert positive clinical value in the early screening of typical symptoms (Figure 3).

#### 3.1.8 Nomogram analysis

**Figure 4:**
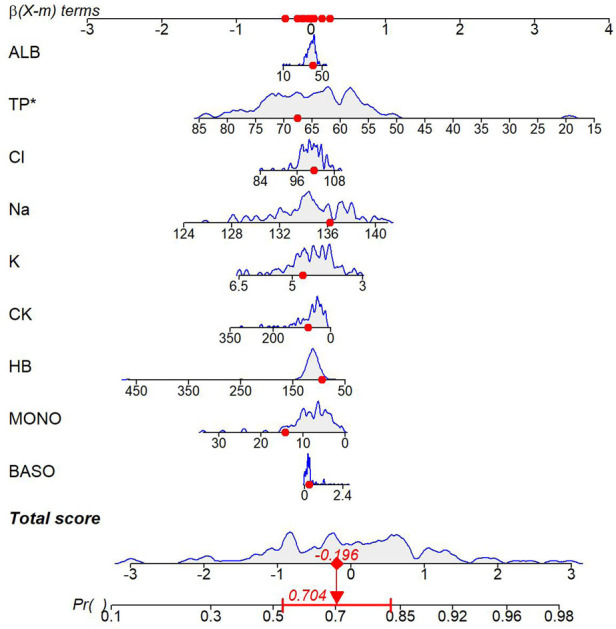
Nomogram of the TKD group and the IKD group.

Based on the multivariate logistic regression model, an individualized prediction nomogram for typical symptoms was constructed. The continuous values of 9 independent variables, including BASO, MONO, HB, CK, K, Na, Cl, TP, and ALB, were transformed into a visual scoring system. Each index corresponded to a respective score according to the measured value. After summing up to obtain the total score, it could be directly mapped to the probability of typical symptoms (predicted probability range: 0.1–0.98), (Figure 4).

### 3.2 Kawasaki coronary artery injury and non-coronary artery injury

#### 3.2.1 Univariate analysis of Kawasaki coronary artery injury and non-coronary artery injury

A total of 131 samples were included, among which 39 samples were in the coronary artery injury group and 92 samples were in the non-coronary artery injury group. The general data and blood test data of the samples are shown in Tables 5, 6 below. After non-parametric independent sample testing, the results indicated that there was no difference in age, height, weight, and BMI between the coronary artery injury group and the non-coronary artery injury group in the general data, while the gender difference was at the critical value. There were significant differences in laboratory indicators Na, TP, ALB, and LDH between the two groups. Compared with the non-coronary artery injury group, the coronary artery injury group had lower Na, lower TP, lower ALB and higher LDH.

**Table 5:** Univariate analysis of coronary artery injury and non-coronary artery injury in Kawasaki.

Indicator	Overall	Non-coronary artery injury (*n* = 92)	Coronary artery injury (*n* = 39)	Statistic	*P*
General information					
Age (m)	34.9 ± 29	33.23 ± 26.53	38.86 ± 34.18	−0.501	0.616
Height (cm)	92.23 ± 22.65	91.62 ± 22.22	93.68 ± 23.85	−0.028	0.978
Weight (Kg)	15.42 ± 12.41	15.87 ± 14.16	14.36 ± 6.71	−0.506	0.613
BMI (kg/m^2^)	16.35 ± 2.37	16.5 ± 2.47	15.98 ± 2.12	−1.460	0.144
Blood routine	15.01 ± 5.9	14.75 ± 6.54	15.62 ± 4.07	−1.485	0.138
WBC (×10^-9^/L)					
NEU%	15.01 ± 5.9	14.75 ± 6.54	15.62 ± 4.07	−1.485	0.138
LYM%	61.38 ± 17.61	62.87 ± 17.72	57.94 ± 17.1	−1.567	0.117
EO%	27.66 ± 14.65	26.77 ± 14.56	29.72 ± 14.81	−1.049	0.294
BASO%	2.45 ± 2.94	2.47 ± 2.6	2.41 ± 3.65	−1.283	0.200
MONO%	0.38 ± 0.47	0.35 ± 0.41	0.44 ± 0.59	−0.264	0.792
PLT (×10^-9^/L)	7.97 ± 5.07	7.41 ± 3.84	9.26 ± 7.05	−0.959	0.337
Hb (g/L)	340.33 ± 109.55	333.59 ± 104.55	355.87 ± 120.27	−0.528	0.597
RBC (×10^-12^/L)	115.56 ± 35.21	117.81 ± 41.6	110.36 ± 9.13	−1.519	0.129
Inflammatory indicators	4.66 ± 3.81	4.88 ± 4.55	4.18 ± 0.27	−0.280	0.780
CRP (mg/L)					
PCT (ng/mL)	72.29 ± 50.56	75.88 ± 50.98	63.81 ± 49.14	−1.367	0.172
Biochemical indicators	1.44 ± 2.46	1.63 ± 2.76	0.99 ± 1.48	−1.787	0.074
ALT (U/L)					
AST (U/L)	45.62 ± 61	47.85 ± 66.29	40.55 ± 47.31	−0.312	0.755
CK (U/L)	48.89 ± 39.06	46.79 ± 36.26	53.64 ± 44.92	−0.678	0.498
K (mmol/L)	68.31 ± 49.53	66.75 ± 51.88	71.83 ± 44.21	−1.371	0.170
Na (mmol/L)	4.47 ± 0.64	4.45 ± 0.65	4.49 ± 0.63	0.741	0.741
Cl (mmol/L)	134.61 ± 2.96	134.98 ± 2.89	133.76 ± 2.99	−2.437	0.015
TP (g/L)	100.35 ± 3.75	100.18 ± 3.92	100.76 ± 3.33	−0.412	0.680
ALB (g/L)	64.4 ± 7.91	65.35 ± 8.85	62.24 ± 4.58	−2.645	0.008
ALP (U/L)	37.63 ± 5.62	39.17 ± 4	34.15 ± 7.09	−4.626	<0.001
LDH (U/L)	193.1 ± 92.72	200.57 ± 106.32	176.21 ± 46.77	−0.970	0.332
Indicator	301.2 ± 76.82	293.26 ± 71.96	319.16 ± 85.1	−2.049	0.040

**Table 6:** Univariate analysis of gender in Kawasaki coronary artery injury vs. non-coronary artery injury.

Gender	Total (%)	Non-coronary artery injury (%)	Coronary artery injury (%)	Statistic	*P*
male	81 (61.83)	52 (56.52)	29 (74.36)	3.693	0.055
female	50 (38.17)	40 (43.48)	10 (25.64)		
Total	131	92	39		

#### 3.2.2 Multivariate logistic regression for Kawasaki coronary artery injury and non-coronary artery injury

The blood test indicators that showed significant differences in the above non-parametric independent sample tests were included in the multivariate logistic regression at one time. The results are as follows, as shown in Table 7: The indicators Na, ALB, and LDH were significant in the multivariate logistic regression and were independent influencing factors of coronary artery injury.

**Table 7:** Multivariate logistic regression analysis of gender in Kawasaki coronary artery injury and non-coronary artery injury.

Indicator	B	Std.Error	Wald	Sig.	Exp(B)	95% CI for EXP (B)	
						lower	Upper
Na	0.036	0.017	4.256	0.039	1.037	1.002	1.073
TP	0.024	0.036	0.432	0.511	1.024	0.954	1.100
ALB	−0.244	0.071	11.695	0.001	0.783	0.681	0.901
LDH	0.006	0.003	4.796	0.029	1.006	1.001	1.012

#### 3.2.3 Model onstruction

Taking coronary artery lesion occurrence as the dependent variable and the above 4 clinically significant laboratory indicators from the difference analysis as independent variables, a multivariate logistic regression model was constructed. The model formula was as follows: Group ∼ Na + TP + ALB + LDH.

#### 3.2.4 Apparent predictive performance of the model

The multivariate logistic regression model constructed in this study showed moderate or higher apparent predictive discrimination for coronary artery lesions. The apparent area under the receiver operating characteristic curve (AUC) was 0.790. The overall predictive accuracy of the model was 0.774, with a sensitivity of 0.395 and a specificity of 0.942, indicating that the model had strong ability to identify negative samples for coronary artery lesions, while its ability to identify positive samples was relatively weak. The apparent calibration of the model reached an ideal state, with a calibration intercept of 0.000 and a calibration slope of 1.000, indicating that the model-predicted probability of coronary artery lesions was highly consistent with the actual occurrence probability in the original dataset. The Hosmer-Lemeshow test showed *P* = 0.188 > 0.05, which further verified the good apparent calibration of the model with no obvious calibration bias. After calibration with 1,000 Bootstrap resampling iterations, the model performance decreased slightly but remained stable overall. The corrected AUC was 0.761, still at a moderate discrimination level. The corrected overall accuracy was 0.753, sensitivity 0.373, and specificity 0.925. All efficacy indexes were slightly lower than the apparent results without a sharp drop, suggesting no serious overfitting and good internal stability. The calibrated calibration indexes shifted slightly, with a calibration intercept of −0.117 and a calibration slope of 0.845. The slope deviated slightly from the ideal value of 1, indicating a minor potential bias in the magnitude of predicted probability in external samples; however, the deviation was small and the overall calibration performance was still acceptable. The detailed values are shown in the Table 8 below:

**Table 8:** Apparent predictive performance of the model in Kawasaki disease patients with and without coronary artery lesions.

Model performance	AUC	Accuracy	Sensitivity	Specificity	Calibration intercept	Calibration slope
Apparent results	0.790	0.774	0.395	0.942	0.000	1.000
Calibrated results	0.761	0.753	0.373	0.925	−0.117	0.845

#### 3.2.5 ROC curve analysis

**Figure 5:**
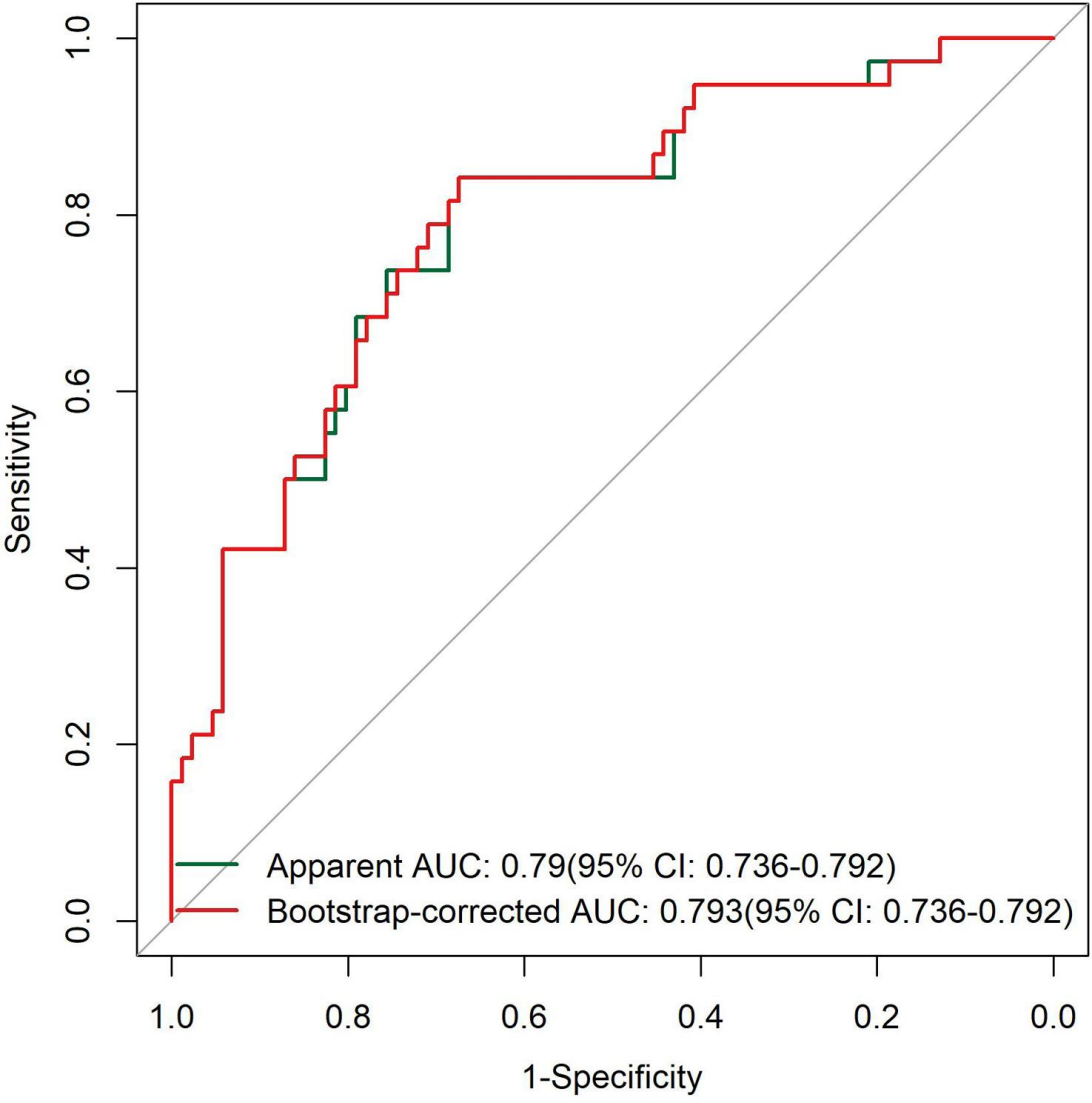
Nomogram for Kawasaki disease patients with and without coronary artery lesions.

The ROC curve results showed that both the apparent AUC and the Bootstrap-corrected AUC of the model were in the range of 0.7–0.8, indicating that the model had moderate discriminatory ability for patients with and without coronary artery lesions. The 95% confidence interval of the AUC was relatively narrow without obvious wide fluctuations, which further verified the statistical reliability of the model's discrimination index and could effectively provide a quantitative reference for risk stratification of coronary artery lesions (Figure 5).

#### 3.2.6 Calibration curve analysis

**Figure 6:**
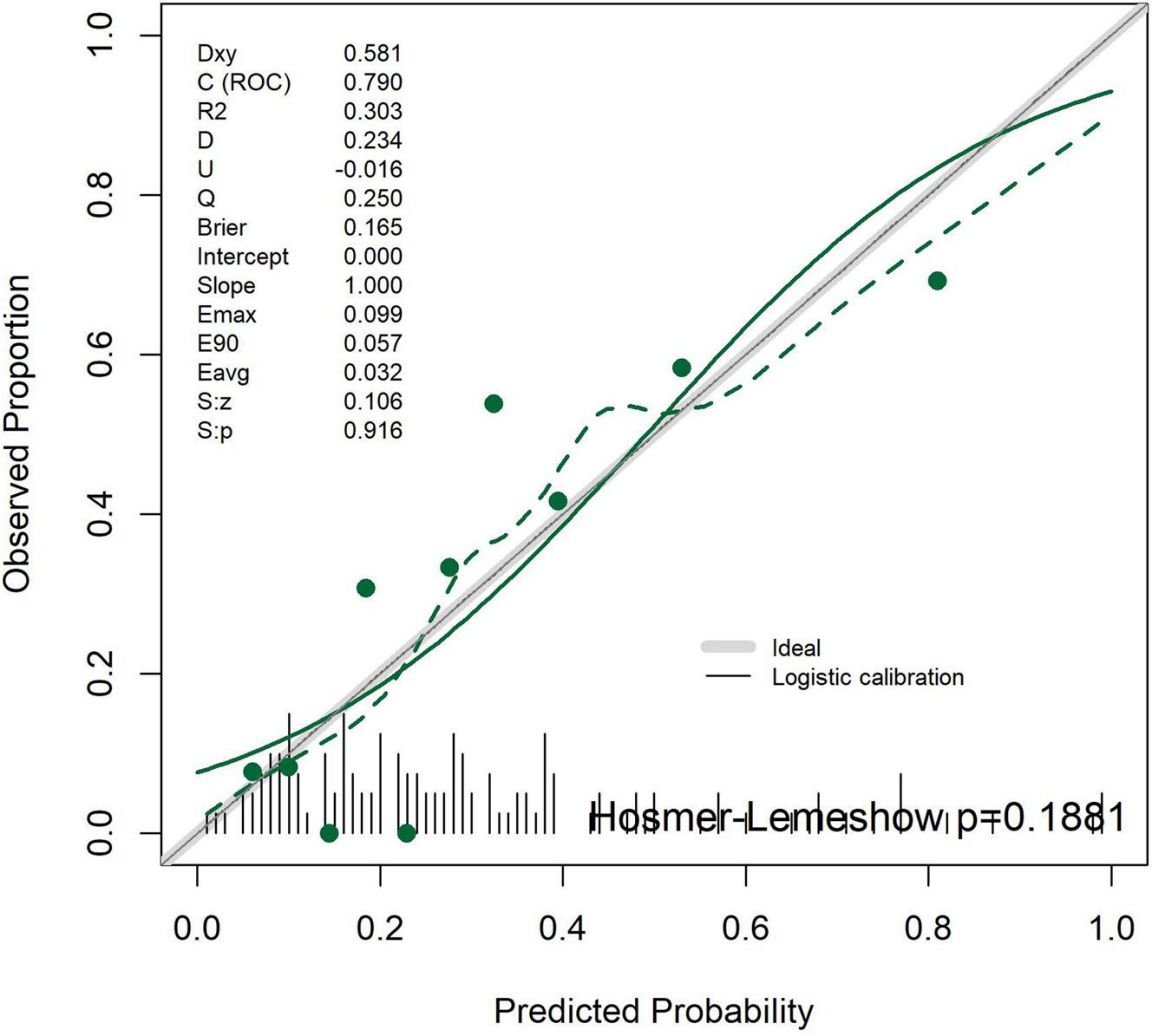
Calibration curves for Kawasaki disease patients with and without coronary artery lesions.

The calibration curve results showed that the logistic calibration line of the model deviated slightly from the ideal reference line (intercept = 0, slope = 1), which was consistent with the results of the Bootstrap-corrected calibration intercept and slope. After grouping the predicted probabilities into equal quantiles, the scatter points of the mean predicted probability and the mean actual disease probability in each group were generally distributed around the ideal line. Combined with the result of the Hosmer-Lemeshow test (*P* > 0.05), it was confirmed that the overall calibration performance of the model was good, the consistency between the predicted probability and the actual occurrence probability of coronary artery lesions was high, and the model's prediction results had reliable practical reference value (Figure 6).

#### 3.2.7 Decision curve analysis

**Figure 7:**
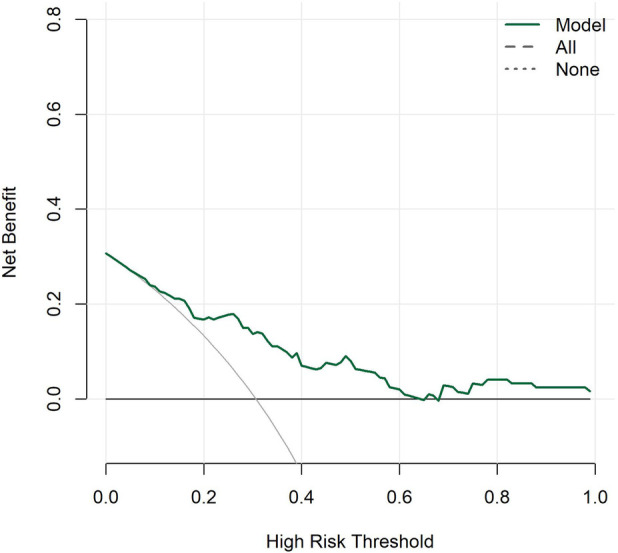
Decision curves for Kawasaki disease patients with and without coronary artery lesions.

The decision curve analysis showed that within most high-risk thresholds for coronary artery lesions, the net benefit of the model constructed in this study was significantly higher than those of the two extreme strategies: "treat all" and "treat none". Moreover, the net benefit curve of the model showed no obvious downward trend, indicating that the application of this model in clinical prediction of coronary artery lesions can effectively provide positive value for clinical decision-making, reduce unnecessary clinical interventions and missed diagnoses, and possess practical clinical significance in risk screening of coronary artery lesions (Figure 7).

#### 3.2.8 Nomogram analysis

**Figure 8:**
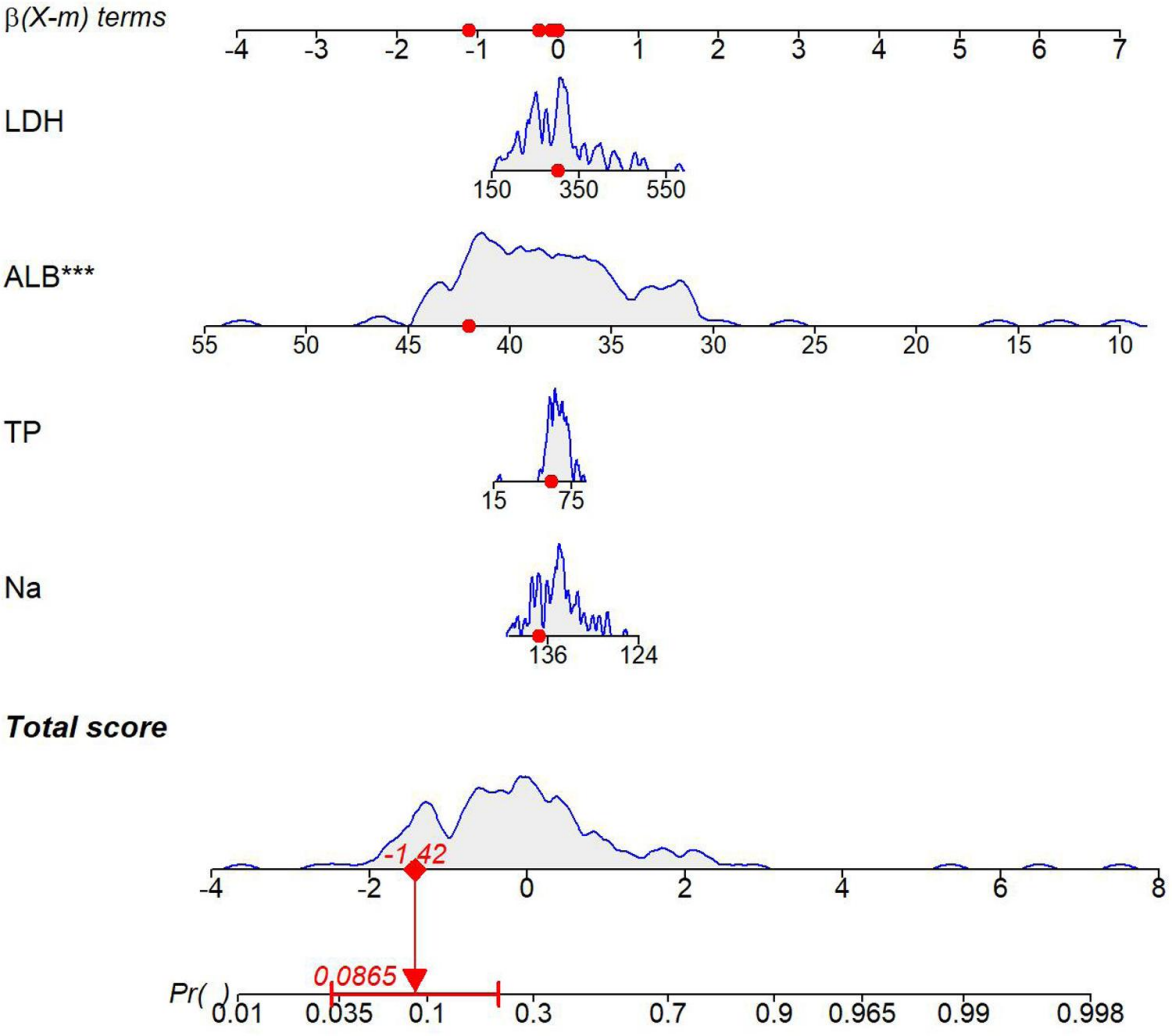
Nomogram for Kawasaki disease patients with and without coronary artery lesions.

Based on the multivariate logistic regression model, an individualized prediction nomogram for coronary artery lesions was constructed. The continuous variables of four indicators, including Na, TP, ALB, and LDH, were transformed into a visual scoring system. Each index corresponded to a respective score according to its measured value. The total score was obtained by summing up the scores of each indicator, which could be directly mapped to the probability of coronary artery lesions (the predicted probability range: 0–0.998) (Figure 8).

## 4 Discussion

### 4.1 Typical Kawasaki disease and incomplete Kawasaki disease

#### 4.1.1 Model performance and clinical translation value

In this study, a multivariate logistic regression model was constructed using 9 statistically significant indicators as independent variables. Internal validation was performed with 1,000 bootstrap resamples to correct for overfitting bias. The results showed that the apparent area under the receiver operating characteristic curve (AUC) of the model was 0.762, with an accuracy of 0.746, sensitivity of 0.930, and specificity of 0.306, indicating that the model had moderate discriminatory ability for typical and incomplete Kawasaki Disease and performed excellently in identifying typical Kawasaki Disease cases. The high sensitivity of the model is of great clinical significance, as it can effectively reduce the false-negative rate and avoid delayed treatment caused by missed diagnosis. After bootstrap correction, the AUC decreased slightly to 0.679 (accuracy 0.699, sensitivity 0.896, specificity 0.237), suggesting mild overfitting, while the model still maintained moderate discrimination and good internal stability. In terms of calibration, the apparent calibration intercept was 0.000 and slope was 1.000, consistent with the ideal state. The Hosmer–Lemeshow test also confirmed good apparent calibration performance. Although the corrected calibration parameters deviated from the ideal values (intercept 0.369, slope 0.524), the scatter points of the calibration curve were generally distributed around the ideal line, indicating acceptable overall calibration reliability.

Decision curve analysis demonstrated that within most risk thresholds for typical Kawasaki Disease, the net benefit of the model was significantly higher than that of the “treat all” and “treat none” strategies. This implies that the model can effectively assist clinicians in identifying high-risk individuals, reduce unnecessary interventions in low-risk patients, and has positive clinical value for early screening of typical Kawasaki Disease. Furthermore, a nomogram was established based on the regression model, converting the continuous values of the 9 indicators into a visual scoring system. Without complex statistical calculations, clinicians can quickly obtain the individualized risk probability of typical Kawasaki Disease by summing the scores corresponding to the measured values, which greatly facilitates the promotion and application of the model in primary pediatric practice.

#### 4.1.2 Clinical analysis of independent risk factors

In this study, through a retrospective analysis of 131 children diagnosed with Kawasaki disease (including 95 cases in the complete group and 36 cases in the incomplete group), we found significant differences in multiple indicators between the two groups. However, the final multivariate logistic regression analysis further confirmed that total protein was the only independent influencing factor for the complete symptoms of Kawasaki disease [Exp (B) = 0.915, 95% confidence interval: 0.854–0.980, *P* = 0.011]. That is, the decrease in serum total protein is an independent risk factor for the appearance of complete symptoms in Kawasaki disease. Total protein is a core component of plasma colloid osmotic pressure, and its level can comprehensively reflect the liver's synthetic function and the systemic inflammatory state. The reason why total protein has independent predictive value is that the vascular endothelial inflammation in complete Kawasaki disease patients is more severe, which not only causes a large amount of protein leakage but also inhibits the liver's synthetic function, ultimately leading to a significant decrease in serum total protein levels (10, 11). This finding provides a specific laboratory target for the clinical differential diagnosis of Kawasaki disease, and total protein detection can also be an important auxiliary reference basis for distinguishing complete from incomplete Kawasaki disease.

### 4.2 Coronary injury and non-coronary injury in Kawasaki disease

#### 4.2.1 Model performance and clinical translation value

This study constructed a multivariate logistic regression model based on four routine clinical test indicators (Na, TP, ALB, LDH), aiming to achieve individualized risk prediction for coronary artery injury. Through internal Bootstrap validation, it was confirmed that the model has moderate discrimination, good calibration, and clinical practicability, and can provide a quantitative reference tool for early risk screening and clinical decision-making of coronary artery injury.

In terms of predictive performance, the apparent AUC of the established model was 0.790, and the bootstrap-corrected AUC was 0.761, both within the moderate discrimination range of 0.7–0.8. These results demonstrated that the model could effectively distinguish patients with coronary artery lesions from those without, and no obvious overfitting was observed, indicating favorable internal stability. The apparent and corrected accuracy were 0.774 and 0.753, respectively. The specificity was excellent (apparent 0.942, corrected 0.925), while the sensitivity was relatively low (apparent 0.395, corrected 0.373). This suggested that the model performed well in identifying negative cases and reducing unnecessary interventions, but had limited ability in detecting positive cases, with a certain risk of false negatives. This may be due to the complex pathogenesis of coronary artery lesions, the limited number of positive cases, or the heterogeneity of the study population. Further optimization by expanding the sample size and incorporating clinical and imaging variables is needed to improve the identification of positive samples.

Calibration is a key indicator of model reliability. The apparent calibration intercept and slope were ideal (0.000 and 1.000), and the Hosmer–Lemeshow test showed *P* = 0.188 (>0.05), indicating high consistency between predicted and actual probabilities. After bootstrap correction, the calibration intercept was −0.117 and the slope was 0.845, with slight but acceptable deviation.

From a clinical perspective, the model and nomogram have obvious practical value. First, the included indicators (Na, TP, ALB, LDH) are routine biochemical tests that are convenient, low-cost, and suitable for primary hospitals and large-scale screening. Second, the nomogram provides a visualized and easy-to-use scoring system, allowing clinicians to quickly estimate the probability of coronary artery lesions without complex calculations. Decision curve analysis confirmed that the model achieved a higher net benefit than treat-all or treat-none strategies across most threshold probabilities. Application of this model can help clinicians make individualized decisions: high-risk patients can undergo further imaging examinations, while low-risk patients can avoid unnecessary tests. This will improve early screening efficiency, optimize medical resource allocation, and reduce the medical burden on patients.

#### 4.2.2 Clinical analysis of independent risk factors

##### 4.2.2.1 Hypoalbuminemia

The most prominent finding of this study is that hypoalbuminemia was the strongest independent predictor of CAL (OR = 0.783, *P* = 0.001). Serum albumin levels were significantly lower in the CAL group (34.15 vs. 39.17 g/L), which is highly consistent with previous studies (12). Kawasaki disease is a systemic vasculitis characterized by massive release of inflammatory cytokines such as TNF-α and IL-6, leading to a marked increase in systemic capillary permeability. As a small-molecular-weight protein, albumin leaks extensively from the intravascular space into the interstitial tissue, and its serum level directly reflects the severity of vascular endothelial injury (13). Since the coronary arteries are also lined with vascular endothelium, severe systemic vascular inflammation inevitably involves the coronary arteries. Therefore, hypoalbuminemia indirectly indicates a high risk of coronary artery involvement. Albumin itself exerts anti-inflammatory, antioxidant, and endothelial function-stabilizing effects. Decreased albumin levels may further weaken the body's endogenous protective mechanisms, creating a vicious cycle that exacerbates vascular injury (14). Thus, a low serum albumin level represents a simple, readily available, and powerful warning sign that warrants high vigilance among clinicians.

##### 4.2.2.2 Hyponatremia

This study is the first to identify hyponatremia as an independent risk factor for CAL by multivariate analysis (OR = 1.037, *P* = 0.039). Serum sodium levels were significantly lower in the CAL group than in the non-CAL group (133.76 vs. 134.98 mmol/L). The underlying mechanisms may involve two aspects: Severe systemic inflammatory responses (e.g., IL-6) can stimulate non-osmotic secretion of antidiuretic hormone, leading to reduced renal water excretion and water retention, thereby causing dilutional hyponatremia (15). Children with Kawasaki disease often present with insufficient water and sodium intake due to fever and poor appetite, which may be further aggravated by concurrent diarrhea or vomiting, resulting in increased sodium loss (16). Hyponatremia indicates a more severe state of inflammation and metabolic disturbance, which may be associated with more extensive vascular lesions.

##### 4.2.2.3 Elevated lactate dehydrogenase

Lactate dehydrogenase (LDH) is a widely distributed intracellular enzyme, and elevated serum LDH generally indicates tissue injury or cell necrosis. This study demonstrated that LDH levels were higher in the CAL group (319.16 vs. 293.26 U/L) and that LDH was an independent predictor in the multivariate model (OR = 1.006, *P* = 0.029). In the context of Kawasaki disease, possible sources of elevated LDH include:Inflammation and microvascular dysfunction in the coronary arteries may lead to myocardial ischemia and hypoxia, resulting in LDH release (17). The inflammatory state in Kawasaki disease may shorten the lifespan of red blood cells, and mild hemolysis can release LDH (18). Hepatic congestion, biliary tract inflammation (e.g., gallbladder hydrops), or direct damage to various tissues and cells caused by systemic inflammation may also contribute to elevated LDH (19). Therefore, elevated LDH reflects the extent and severity of multi-organ tissue injury induced by Kawasaki disease, and its level parallels the risk of coronary artery lesions.

##### 4.2.2.4 Trend of sex

The proportion of male patients was higher in the CAL group (74.36% vs. 56.52%, *P* = 0.055). Although this difference did not reach statistical significance, the trend was consistent with numerous epidemiological observations worldwide that male sex is a risk factor for CAL (1).

## 5 Limitations of the study

This study has several limitations. First, as a single-center retrospective study, selection bias was inevitable, limiting the generalizability of the results. In addition, the completeness of medical records may have affected the accuracy of the findings. Second, the sample size was relatively small with a low proportion of positive cases, resulting in insufficient statistical power that may influence model fitting. Third, only blood test indicators were included in the models, without integrating clinical symptoms, imaging findings, and other multidimensional information, making it difficult to comprehensively reflect the characteristics of the disease. Fourth, only internal validation using Bootstrap was performed, and multicenter external validation was lacking, providing insufficient evidence for clinical application and promotion. Fifth, it is a pity that dynamic changes in laboratory parameters were not analyzed.

## 6 Conclusions

In conclusion, this study established multivariate logistic regression prediction models based on routine clinical laboratory indicators for complete vs. incomplete Kawasaki disease and coronary artery lesions (CAL) vs. non-CAL, respectively. After Bootstrap internal validation, both models showed moderate discrimination, satisfactory calibration, and good clinical practicability. The accompanying nomograms allow individualized quantitative risk prediction, providing a convenient quantitative tool for early screening, differential diagnosis, and clinical decision-making in primary care settings. Total protein was an independent factor for complete Kawasaki disease, while albumin, serum sodium, and lactate dehydrogenase were independent risk factors for CAL, providing specific laboratory targets for risk stratification of Kawasaki disease. Although this study has limitations such as a single-center design and limited sample size, it provides a novel strategy for the development of clinical prediction models for Kawasaki disease. After further multicenter validation and model optimization, the models are expected to have improved clinical application value.

## Data Availability

The original contributions presented in the study are publicly available. This data can be found here: https://doi.org/10.5281/zenodo.19464813. Further inquiries can be directed to the corresponding author/s.
