# Correction: A predictive model and nomogram for coronary artery injury in kawasaki disease based on laboratory indicators: a retrospective study

**DOI:** 10.3389/fped.2026.1940030

**Published:** 2026-07-27

**Authors:** Yanyan Li, Zhiqing Chen, Xiaoyan Wang, Chaolong Zheng, Ziyang Cui, Sisi Cheng, Limin Chu, Changjun Ren, Guiling Liu

**Affiliations:** Department of Pediatrics, The First Hospital of Hebei Medical University, Shijiazhuang, China

**Keywords:** coronary artery lesion, Kawasaki disease, laboratory parameters, nomogram, predictive model

Incorrect funding

Adding

The funder “The Medical Science Research Project of Hebei Provincial Health Commission, No. 20231035” to Yanyan Li was erroneously omitted.

The original version of this article has been updated.

